# Organocatalyzed Intramolecular Carbonyl-Ene Reactions

**DOI:** 10.3390/molecules21060713

**Published:** 2016-05-31

**Authors:** Heidi A. Dahlmann, Amanda J. McKinney, Maria P. Santos, Lindsey O. Davis

**Affiliations:** Department of Chemistry and Biochemistry, Berry College, P.O. Box 495016, Mt. Berry, GA 30149, USA; hdahlmann@berry.edu (H.A.D.); amanda.mckinney@vikings.berry.edu (A.J.M.); maria.santos@vikings.berry.edu (M.P.S.)

**Keywords:** organocatalysis, carbonyl-ene, phosphoric acid, *N*-triflylphosphoramide

## Abstract

An organocatalyzed intramolecular carbonyl-ene reaction was developed to produce carbocyclic and heterocyclic 5- and 6-membered rings from a citronellal-derived trifluoroketone and a variety of aldehydes. A phosphoramide derivative was found to promote the cyclization of the trifluoroketone, whereas a less acidic phosphoric acid proved to be a superior catalyst for the aldehyde substrates.

## 1. Introduction

The carbonyl-ene reaction is a well-studied transformation in organic chemistry, as it affords an atom-economical method for synthesizing homoallylic alcohols [1]. Traditionally, Lewis acids have been used to catalyze this reaction [2,3], but organocatalysis has recently emerged as a powerful means for facilitating many organic transformations [4,5,6], including carbonyl-ene reactions. Clarke and co-workers developed the first organocatalyzed carbonyl-ene reaction using the Schreiner catalyst [7], a thiourea derivative [8]. An asymmetric variant was then developed by Rueping *et al.* using a chiral *N*-triflylphosphoramide [9]. The reaction yielded α-hydroxyesters in good yield and enantioselectivity, but the scope was limited to intermolecular reactions and required the use of an activated enophile. Recently, List and co-workers reported an intramolecular carbonyl-ene cyclization to afford pyrrolidines, tetrahydrofurans, and cyclopentanes using a chiral imidodiphosphate catalyst [10]. While this report serves as a hallmark for Brønsted-acid-catalyzed intramolecular carbonyl-ene reactions, the scope was limited to the formation of the kinetically-favored five-membered rings [11]. The majority of these products were pyrrolidines derived from *N*-tosylated aminoaldehyde, the parent molecule which was known to spontaneously undergo intramolecular carbonyl-ene cyclization [12], while less activated substrates required up to 11 days to reach completion. Noting the utility of this reaction, but also the limitations of current reports, we set out to develop a Brønsted-acid-catalyzed intramolecular carbonyl-ene reaction with a complementary substrate scope and faster reaction times. Herein, we describe organocatalyzed intramolecular carbonyl-ene reactions that produced carbocyclic and heterocyclic 5- and 6-membered rings.

## 2. Results and Discussion

**Figure 1:**
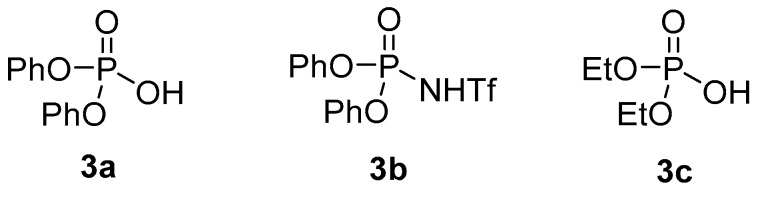
Brønsted acids screened for carbonyl-ene reaction.

We began our investigation by screening a variety of Brønsted acids for their ability to cyclize citronellal-derived trifluoromethylketone **1** (Table 1), selecting this activated substrate based on previous reports of trifluoropyruvate derivatives serving as carbonyl-acceptors in intermolecular carbonyl-ene reactions [8,9]. Simple Brønsted acid catalysts such as H_3_PO_4_ and HCl were unable to catalyze the reaction at an acceptable rate, producing little to no product within 24 h (Table 1, Entries 1 and 2) [13]. Similarly, the phosphoric acid derivative diphenyl phosphate (Figure 1, **3a**), induced very slow conversion of substrate, resulting in a low yield of ene product (Table 1, Entry 3). In contrast, the more acidic *N*-triflyl phosphoric amide **3b** (Figure 1) catalyzed the reaction at a significantly higher rate, resulting in complete conversion and good yields in as few as 7 h (Table 1, Entries 4 and 8) [14]. Notably, we were able to decrease the catalyst loading from 0.5 to 0.2 equivalents without a significant loss in yield (Table 1, Entries 4 and 5). Decreasing the concentration of the reaction resulted in a longer reaction time with a small drop in yield (Table 1, Entries 6 and 7).

Concurrently, we screened Brønsted acids for their ability to catalyze the cyclization of citronellal (**4a**). While citronellal is less activated than the corresponding trifluoromethyl ketone, it serves as the prototypical substrate for a Type I carbonyl-ene cyclization, as it is commercially available [15,16,17]. Surprisingly, the use of phosphoramide **3b** resulted in the isolation of a complex mixture of products with only a trace yield of ene product **5a** and no starting material recovery (Table 2, Entry 1). Diethyl phosphate (**3c**) successfully promoted the reaction, albeit slowly, resulting in a low yield and a 77% recovery of starting material after 24 h (Table 2, Entry 2). We were pleased to find a significant increase in reaction rate and yield after 24 h when phosphoric acid derivative **3a** was used as a catalyst. Under these mild reaction conditions [18], isopulegol (**5a**) was the primary diastereomer isolated from the reaction mixture in addition to a small amount of neoisopulegol (**5a′**), typically in a 2:1 ratio (Entries 3–5). A similar yield of product **5a** was obtained when only 0.06 equivalents of catalyst were used at a higher concentration (0.5 M) compared to 0.5 equivalents at 0.1 M, but a decreased selectivity was observed in the isolated products (compare Entries 3 and 4) [19]. At an even higher substrate concentration (2 M), additional uncharacterized products were formed and the yield of **5a** decreased considerably (Table 2, Entry 5).

Once the cyclization of the aldehyde substrate was optimized (0.5 equiv of **3a**, 0.1 M, 24 h, rt), the scope of the reaction was explored. The carbonyl-ene reaction proceeds with excellent yield to give 3,4-disubstituted piperidine product **5b** (Table 3, Entry 1), favoring the *trans* diastereomer. Aryl aldehyde **4c**, which required a full equivalent of **3a** to undergo complete conversion within 24 h, afforded a moderate yield of the carbonyl-ene product **5c** (again with the *trans* product favored over the *cis* product) as well as a substantial amount (~17%) of the conjugated diene, 3-isopropenyl-1,2-dihydronaphthalene, that resulted from an elimination reaction (Table 3, Entry 2). Lastly, the cyclization of commercially available 2,6-dimethyl-5-heptenal (**4d**) resulted in the formation of five-membered ring **5d** with great diastereoselectivity, albeit in only moderate yield (Entry 3). The reaction conditions have been modified in an attempt to increase the yield of the carbonyl-ene product; however, in each case, a complex mixture of products was isolated with no starting material recovered.

## 3. Materials and Methods

### 3.1. General

Citronellal (93%) was obtained from Acros and was purified with normal-phase column chromatography. 2,4-dimethylheptenal (80%) was purchased from Aldrich and was purified via normal phase chromatography before use. Diphenyl phosphate (**3a**) was purchased from Aldrich. Trifluoromethyl ketone **1** was prepared from citronellic acid as previously described [20]. Diphenylphosphoramide (**3b**) was prepared as previously described [21]; following chromatographic purification, catalyst **3b** was washed with 6 M of HCl and extracted with chloroform to ensure protonation of the catalyst, as discussed for the preparation of related *N*-triflylphosphoramide catalysts [22]. 2-(4-methyl-3-pentenyl)benzaldehyde [23] and 4-methyl-*N*-(3-methylbut-2-enyl)-*N*-(3-oxopropyl)benzenesulfonamide [24] were prepared as previously described. Anhydrous dichloromethane was obtained from a solvent system purchased by Pure Process Technology. Normal-phase flash-chromatography was carried out manually on silica gel (Mallinckrodt Chemicals, 60 Å, 40–63 micron) or with a Combi-flash MPLC system equipped with Redi-Sep Gold chromatography cartridges. ^1^H-NMR spectra were obtained by using a Jeol 400 MHz spectrometer (Jeol USA, Inc., Peabody, MA, USA). Chemical shifts are reported in parts per million relative to TMS. Coupling constants were reported in Hertz, and multiplicities were indicated using the following symbols: s (singlet), d (doublet), t (triplet), q (quartet), m (multiplet), ddd (doublet of doublets of doublets), *etc.* ^13^C-NMR data was obtained using Jeol 400 MHz NMR operating at 100 MHz. All products were characterized by ^1^H-NMR and ^13^C-NMR and compared with available literature data. High-resolution mass spectra (HRMS) of **2a**/**a′** were obtained on a Thermo LTQ-FTMS instrument (ThermoFisher Scientific, Waltham, MA, USA).

### 3.2. General Procedure for Intramolecular Carbonyl-Ene Reactions

Aldehyde or CF_3_-ketone substrate (0.4–2 mmol), catalyst **3** (0.1–1 equivalents), and anhydrous dichloromethane (0.1–2 M with respect to aldehyde) were added to a small glass vial containing a stir bar. After stirring at room temperature for 24–48 h, the reaction was concentrated and purified by column chromatography.

#### 3.2.1. Synthesis of Compounds **2a**/**a′**

Compounds **2a**/**a′** were prepared according to the above-described general procedure by stirring CF_3_ ketone **1** (208 mg, 1 mmol) and diphenylphosphoramide **3b** (76 mg, 0.2 mmol) in anhydrous dichloromethane (0.5 mL) at room temperature for 24 h to provide a mixture of diastereomers as a colorless oil in 86% yield (flash-chromatography: 20% diethyl ether in petroleum ether). Retention factor of **2a**/**2a′** = 0.3 (5% ethyl acetate: 95% hexanes). The relative stereochemical assignments of **2a**/**2a′** were made on the basis of coupling constants for H2 and H6_ax_ [25]. For **2a**, the coupling constants for H2 = 3.6 and 12.8 Hz indicated axial orientation, and the coupling constants for H6_ax_ = 12.4 and 14.0 Hz indicated axial-axial splitting with H5 and germinal coupling. Therefore, H5 must be in the axial position and *trans* to H2. For **2a′**, H2 had a coupling constant of 13.2 Hz, indicating axial orientation, and one of the coupling constants for H6_ax_ = 4.5 Hz indicated axial-equatorial splitting with H5. Therefore, H5 must be in the equatorial position and *cis* to H2. ^1^H-NMR of **2a** (400 MHz, CDCl_3_): δ 4.94 (s, 1H, vinylic H); 4.83 (s, 1H, vinylic H); 2.37 (s, 1H, -OH); 2.25 (dd, *J =* 3.7, 12.7 Hz, 1H, H2); 1.96 (ddd, *J* = 1.8, 3.2, 13.9 Hz, 1H, H6_eq_); 1.84 (m, 1H, H5_eq_); 1.83 (s, 3H, vinylic Me); 1.80 (m, 1H, H3_eq_); 1.73 (m, 1H, H4_eq_); 1.55 (m, 1H, H3_ax_); 1.18 (ddd, *J* = 1.9, 12.4, 13.9, H6_ax_); 0.94 (m, 1H, H4_ax_); 0.93 (d, *J* = 8.0 Hz, 3H, C5-Me). ^13^C-NMR (100 MHz): δ 147.6 (vinylic C); 126.1 (q, *J* = 290 Hz, -CF_3_); 112.2 (vinylic C); 74.3 (q, *J* = 26 Hz, C1); 46.3 (C2); 39.0 (C6); 34.3 (C4); 28.5 (C3); 26.5 (C5); 24.2 (vinylic Me); 21.9 (C5-Me). HRMS, APCI: Calcd. for C_11_H_17_F_3_O (M+): 222.12315; found: 223.13509. ^1^H-NMR of **2a′** (400 MHz): δ 5.01 (m, 1H, vinylic H); 4.90 (m, 1H, vinylic H); 2.92 (s, 1H, -OH); 2.31 (m, 1H, H2); 2.25 (ddd, *J* = 1.9, 3.4, 13.6 Hz, 1H, H6_eq_); 1.89 (m, 1H, H3_eq_); 1.82 (m, 1H, H4_eq_); 1.75 (s, 3H, vinylic Me); 1.73 (m, 1H, H5); 1.66 (m, 1H, H3_ax_); 1.125 (tq, *J* = 2.4, 2.4, 2.4, 13.6, 13.6 Hz, H6); 1.00 (m, 1H, H4_ax_); 0.94 (d, *J* = 6.5 Hz, 3H, C5-Me). ^13^C-NMR (100 MHz, CDCl_3_): δ 144.1 (vinylic C); 126.6 (q, *J* = 286 Hz, -CF_3_); 115.7 (vinylic C); 73.0 (q, *J* = 27 Hz, C1); 53.2 (C2); 42.2 (C6); 34.1 (C4); 28.2 (C5); 26.3 (C3); 22.4 (C5-Me); 19.7 (vinylic Me). HRMS, ESI: Calcd. for C_11_H_18_F_3_O^+^ (M + H^+^): 223.13043; found: 223.13041.

#### 3.2.2. Synthesis of Compounds **5a**/**a′**

Compounds **5a**/**a′** were prepared according to the above-described general procedure by stirring citronellal (101.5 mg, 0.658 mmol) and diphenyl phosphate (**3a**) (82.0 mg, 0.328 mmol) in anhydrous dichloromethane (6.6 mL) at room temperature for 22 h to provide a colorless oil in a 61% yield (products were purified by flash-chromatography with 10% diethyl ether in petroleum ether). The NMR spectra of isopulegol (**5a**) and neoisopulegol (**5a′**) matched those previously reported [26]. ^1^H-NMR of isopulegol ((1α, 2α,5β)-5-methyl-2-(1-methylethenyl)cyclohexanol, **5a**) (400 MHz, CDCl_3_): δ 4.91 (m, 1H); 4.86 (br s, 1H); 3.46 (dt, *J* = 4.3, 10.4, 10.4 Hz, 1H); 2.04 (m, 1H); 1.89 (ddd, *J* = 3.4, 10.0, 12.8 Hz, 1H); 1.71 (d, *J* = 1.0 Hz, 3H); 1.65 (m, 2H); 1.48 (m, 2H); 1.34 (dt, *J* = 3.4, 12.4, 12.4 Hz, 1H); 0.96 (m, 2H); 0.95 (d, *J* = 6.5 Hz, 3H). ^1^H-NMR of neoisopulegol ((1α, 2α, 5β)-5-methyl-2-(1-methylethenyl)cyclohexanol, **5a′**) (400 MHz, CDCl_3_): δ 4.95 (br s, 1H); 4.78 (br s, 1H); 3.98 (m, 1H); 1.98 (m, 2H); 1.79 (s, 3H); 1.73 (m, 2H); 1.55 (br s, 1H); 1.45 (m, 1H); 1.12 (m, 1H); 0.95 (m, 2H); 0.88 (d, *J* = 6.4 Hz, 3H).

#### 3.2.3. Synthesis of Compounds **5b**/**b′**

Compounds **5b/b′** were prepared according to the above-described general procedure by using 4-methyl-*N*-(3-methylbu-2-enyl)-*N*-(3-oxopropyl)benzenesulfonamide (106.3 mg, 0.378 mmol) and diphenyl phosphate (**3a**) (48 mg, 0.192 mmol) in anhydrous dichloromethane (3.8 mL) at room temperature for 24 h to provide an oil in a 89% yield (products were purified by flash-chromatography with a stepwise gradient of 20%–30% ethyl acetate in hexanes). The NMR spectra of isolated piperidines matched those previously reported [13]. ^1^H-NMR of *trans*-3-isopropenyl-1-(toluene-4-sulfonyl)piperidin-4-ol, **5b**, (400 MHz, CDCl_3_): δ 7.64 (d, *J* = 8.0 Hz, 2H); 7.32 (d, *J* = 8.0 Hz, 2H); 5.01 (s, 1H); 4.89 (s, 1H); 3.84 (m, 1H); 3.76 (m, 1H); 3.44 (dt, *J* = 4.5, 10.1, 10.1 Hz, 1H); 2.44 (s, 3H); 2.37 (dt, *J* = 2.8, 12.4, 12.4 Hz, 1H); 2.26 (dt, *J* = 3.4, 11, 11 Hz, 1H); 2.17 (t, *J* = 11 Hz); 2.04 (m, 1H); 1.71 (s, 3H); 1.64 (m, 1H). ^1^H-NMR of *cis*-3-isopropenyl-1-(toluene-4-sulfonyl)piperidin-4-ol, **5b′** (400 MHz, CDCl_3_): δ 7.66 (d, *J* = 8.1 Hz, 2H); 7.32 (d, *J* = 8.1 Hz, 2H); 4.99 (s, 1H); 4.59 (s, 1H); 3.97 (m, 1H); 3.59 (m, 2H); 2.60 (dt, *J* = 3.1, 12, 12 Hz, 1H); 2.57 (t, *J* = 11.5 Hz, 1H); 2.42 (s, 3H); 2.37 (d, *J* = 12.1 Hz, 1H); 1.96 (dq, *J* = 2.8, 2.8, 2.8, 13.9 Hz, 1H); 1.87 (m, 1H); 1.77 (s, 3H).

#### 3.2.4. Synthesis of Compounds **5c**/**c′**

Compounds **5c**/**c′** were prepared according to the above-described general procedure by using 2-(4-methyl-3-pentenyl)benzaldehyde (123.6 mg, 0.656 mmol) and diphenyl phosphate (**3a**) (183.0 mg, 0.656 mmol) in anhydrous dichloromethane (7.3 mL) at room temperature for 24 h to provide a mixture of diastereomers in a 44% yield (products were purified by flash-chromatography with a stepwise gradient of 0%–1% diethyl ether in petroleum ether). The NMR spectra of alcohol products (**5c** and **5c′**) [27], and the elimination product [28] matched those previously reported. ^1^H-NMR of *trans*-2-isoprenyl-1,2,3,4-tetrahydro-1-naphthalenol, **5c**, (400 MHz, CDCl_3_): δ 7.61 (d, *J* = 7.6 Hz, 1H); 7.23 (m, 1H); 7.19 (m, 1H); 7.09 (d, *J* = 7.2 Hz, 1H); 4.96 (m, 1H); 4.89 (m, 1H); 4.70 (d, *J* = 9.5 Hz, 1H); 2.89 (ddd, *J* = 5.5, 11.2, 16.7 Hz, 1H); 2.82 (ddd, *J* = 3.2, 5.4, 16.7 Hz, 1H); 2.39 (ddd, *J* = 3.1, 9.5, 11.8 Hz, 1H); 2.07 (br s, 1H); 1.93 (m, 1H); 1.83 (m, 1H); 1.81 (s, 3H). ^1^H-NMR of *cis*-2-isoprenyl-1,2,3,4-tetrahydro-1-naphthalenol, **5c′** (400 MHz, CDCl_3_): δ 7.38 (dd, *J* = 2.1, 7.0 Hz, 1H); 7.23 (m, 1H); 7.22 (m, 1H); 7.16 (m, 1H); 5.08 (m, 1H); 4.91 (m, 1H); 4.76 (m, 1H); 2.95 (ddd, *J* = 2.0, 5.4, 17.0 Hz, 1H); 2.81 (ddd, *J* = 5.9, 12.3, 17.0 Hz, 1H); 2.40 (m, 1H); 2.06 (dq, *J* = 5.4, 12.7, 12.7, 12.7 Hz,1H); 1.91 (s, 3H); 1.79 (m, 2H). ^1^H-NMR of 3-isopropenyl-1,2-dihydronaphthalene (400 MHz, CDCl_3_): δ 7.11 (m, 4H); 6.57 (s, 1H); 5.22 (s, 1H); 5.04 (s, 1H); 2.85 (t, *J* = 8 Hz, 2H); 2.53 (t, *J* = 8 Hz, 2H); 2.05 (s, 3H).

#### 3.2.5. Synthesis of Compounds **5d**/**d′**

Compounds **5d**/**d′** were prepared according to the above-described general procedure by using 2, 6-dimethyl-5-heptenal (280 mg, 2 mmol) and diphenyl phosphate (**3a**) (250 mg, 1 mmol) in anhydrous dichloromethane (20 mL) at room temperature for 19 h to provide a mixture of diastereomers in a 37% yield (products were purified by flash-chromatography with a stepwise gradient of 5–20% diethyl ether in petroleum ether). ^1^H-NMR of **5d** (major isomer), (400 MHz, CDCl_3_): δ 4.81 (d, *J* = 6.2 Hz, 2H); 3.4 (t, *J* = 9.3 Hz, 1H); 2.38 (q, *J* = 9.3 Hz, 1H); 1.78–1.95 (m, 3H); 1.74 (bs, 1H, -OH); 1.73 (s, 3H); 1.5 (m, 1H); 1.15 (m, 1H); 1.08 (d, *J* = 6.5 Hz, 3H). ^13^C-NMR (100 MHz): δ 146.3, 110.8, 81.8, 55.0, 41.0, 29.3, 26.0, 19.7, 18.1. The NMR spectra of **5d**/**d′** match those previously reported for an analogous compound [29]. The protons of **5d′** overlapped with the major isomer (**5d**) except for H1, which appeared at 3.93 ppm, and the β-methyl group at 1.00 ppm.

NMR spectra for products **2a**/**a′** and **5d**/**d′** can be found in the Supplementary Materials.

## 4. Conclusions

In summary, we have reported organocatalyzed intramolecular carbonyl-ene cyclizations of a citronellal-derived trifluoroketone and several aldehydes. The scope of this reaction is more general than previous reports and produces various *trans*–configured carbocyclic and heterocyclic 5- and 6-membered rings in moderate-to-good yield. In addition, these reactions are complete within 7–24 h. Further exploration of the scope of the reaction as well as screening enantioselective catalysts are ongoing in our laboratory.

## Figures and Tables

**Table 1:** Optimization of carbonyl-ene cyclization of trifluoroketone **1**. 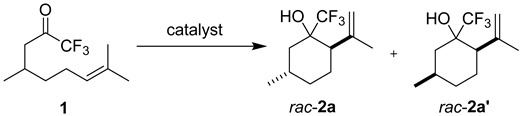

Entry ^1^	Catalyst	Equiv. of Catalyst	[1] (M)	*t* (h)	Yield 2 ^2^ (%)	d.r. (2a:2a′) ^3^
1	H_3_PO_4_	1	2	24	0	-
2	HCl	1	2	24	13	-
3	**3a**	0.5	2	24	25	2:1
4	**3b**	0.5	2	24	84	2:1
5	**3b**	0.2	2	24	86	2:1
6	**3b**	0.1	2	24	69	2.1:1
7	**3b**	0.1	0.5	48	75	2.1:1
8	**3b**	0.5	2	7	79	2.2:1

^1^ All reactions were run in anhydrous dichloromethane at 25 °C. ^2^ The yield is reported as a mixture of diastereomers **2a**/**2a′**; however, products **2a** and **2a′** can be separated using column chromatography (see Material and Methods Section). ^3^ The diastereomeric ratios (**2a**:**2a′**) were determined using ^1^H-NMR integration of isolated products.

**Table 2:** Optimization of carbonyl-ene cyclization of citronellal **4a**. 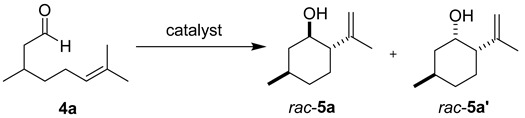

Entry ^1^	Catalyst	Equiv. of Catalyst	[4a] (M)	*t* (h)	Yield 5a ^2^ (%)	d.r. (5a:5a′) ^3^
1	**3b**	1	0.1	24	trace	-
2	**3c**	0.5	0.1	24	14	1:1
3	**3a**	0.5	0.1	22	61	2.4:1
4	**3a**	0.06	0.6	24	65	1.5:1
5	**3a**	0.1	2	18	31	2:1

^1^ All reactions were run in anhydrous dichloromethane at 25 °C. ^2^ The yield is reported as a mixture of diastereomers **5a**/**5a′**; however, products **5a** and **5a′** can be separated using column chromatography (see Material and Methods section). ^3^ The diastereomeric ratios (**5a**:**5a′**) were determined using ^1^H-NMR integration of isolated products.

**Table 3:** Scope of the carbonyl-ene reaction of aldehydes.

Entry ^1^	Aldehyde	Product	Yield (%) (d.r.) ^2,3^
1	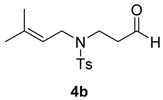	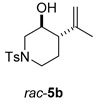	89 (2.7:1)
2 ^4^	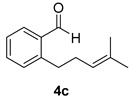	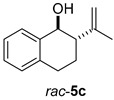	44 (3.3:1)
3	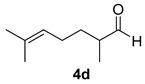	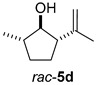	37 (>10:1)

^1^ Unless otherwise noted, reactions were run in anhydrous dichloromethane at 25 °C for 19–24 h with [**4**] = 0.1 M and 0.5 equivalents of **3a**. ^2^ The yield is reported as a mixture of diastereomers **5**/**5′**; however, the diastereomeric products of **5b** and **5c** can be separated using column chromatography (see Material and Methods section). ^3^ The diastereomeric ratios (*trans*:*cis*; **5**:**5′**) were determined using ^1^H-NMR integration of isolated products. The methyl group of the minor diastereomer **5d′** is *cis* to the hydroxyl group. ^4^ One molar equivalent of **3a** was used.

## Supplementary Materials

Supplementary materials can be accessed at: http://www.mdpi.com/1420-3049/21/6/713/s1.
