# Supplementary material for: Organocatalyzed Intramolecular Carbonyl-Ene Reactions
Source: Molecules. 2016 May 31;21(6):713. doi: 10.3390/molecules21060713 (PMC6272844; doi:10.3390/molecules21060713)
Supplement: Supplementary file 1 [file molecules-21-00713-s001.pdf]

Supplementary Materials: Organocatalyzed Intramolecular Carbonyl-Ene Reactions

Heidi A. Dahlmann, Amanda J. McKinney, Maria P. Santos and Lindsey O. Davis

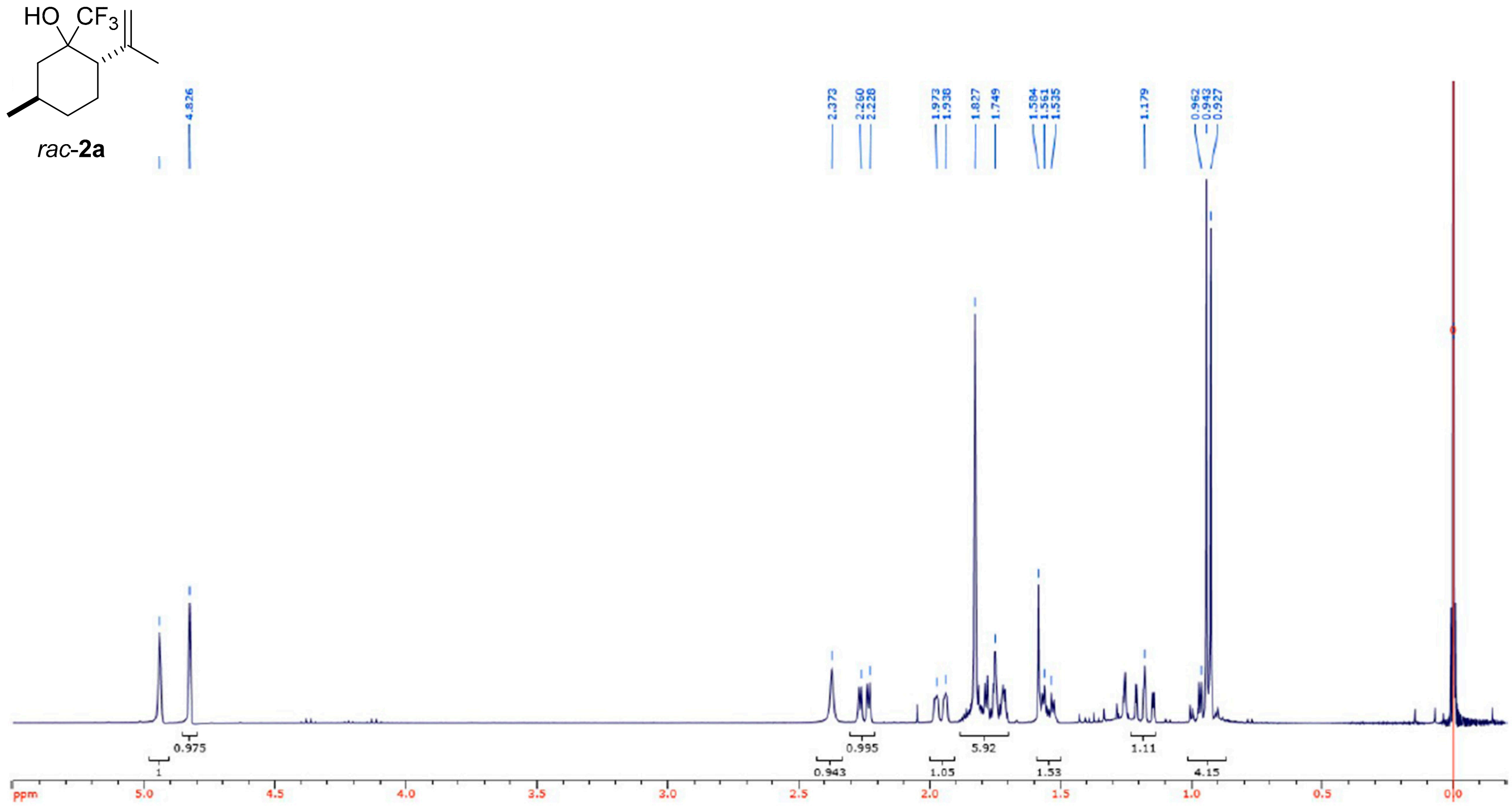

Figure S1. ^{1}H-NMR of **2a**.

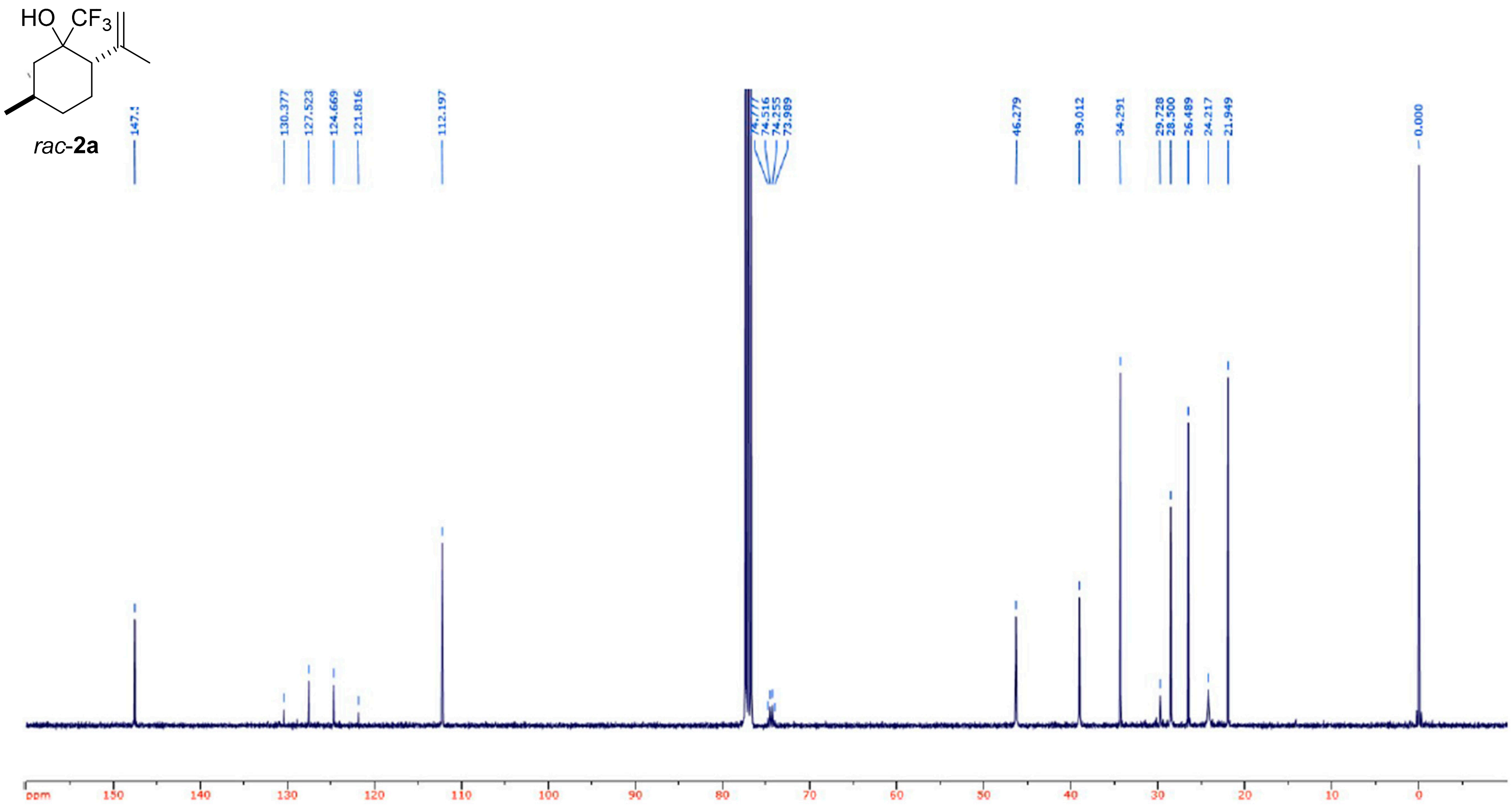

Figure S2. ^{13}C-NMR of **2a**.

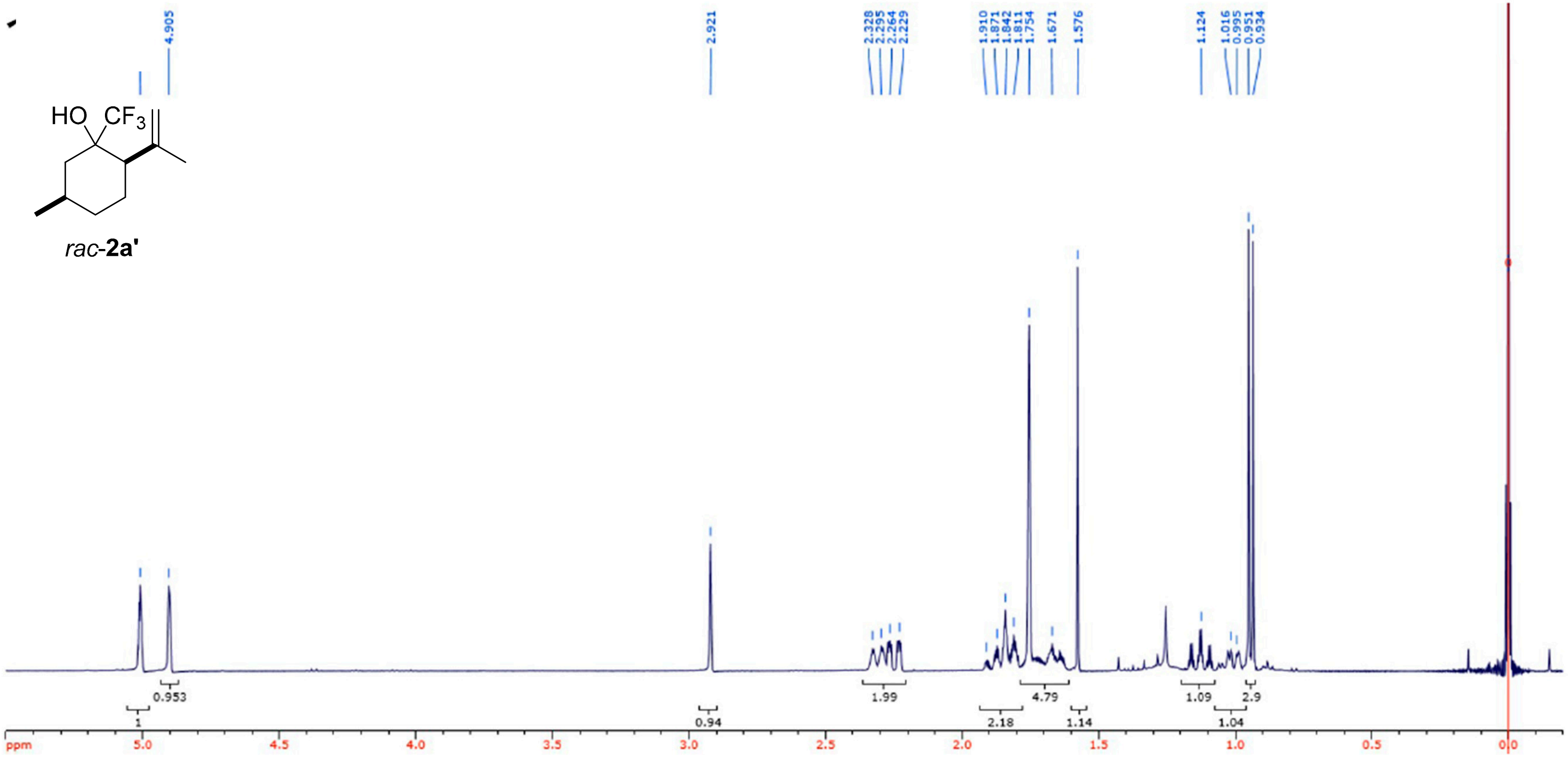

Figure S3. ^{1}H-NMR of **2a**′.

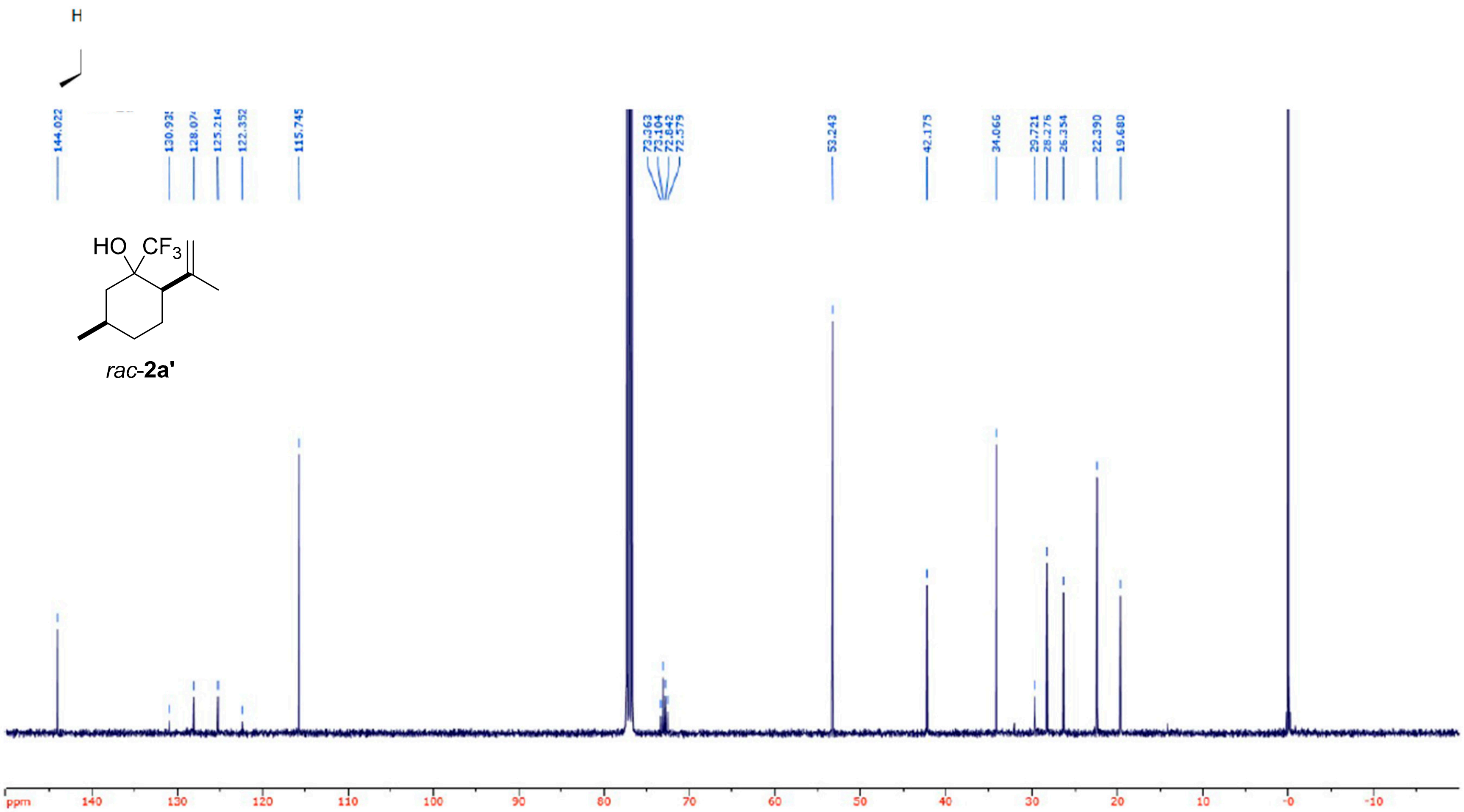

Figure S4. ^{13}C-NMR of **2a**′.

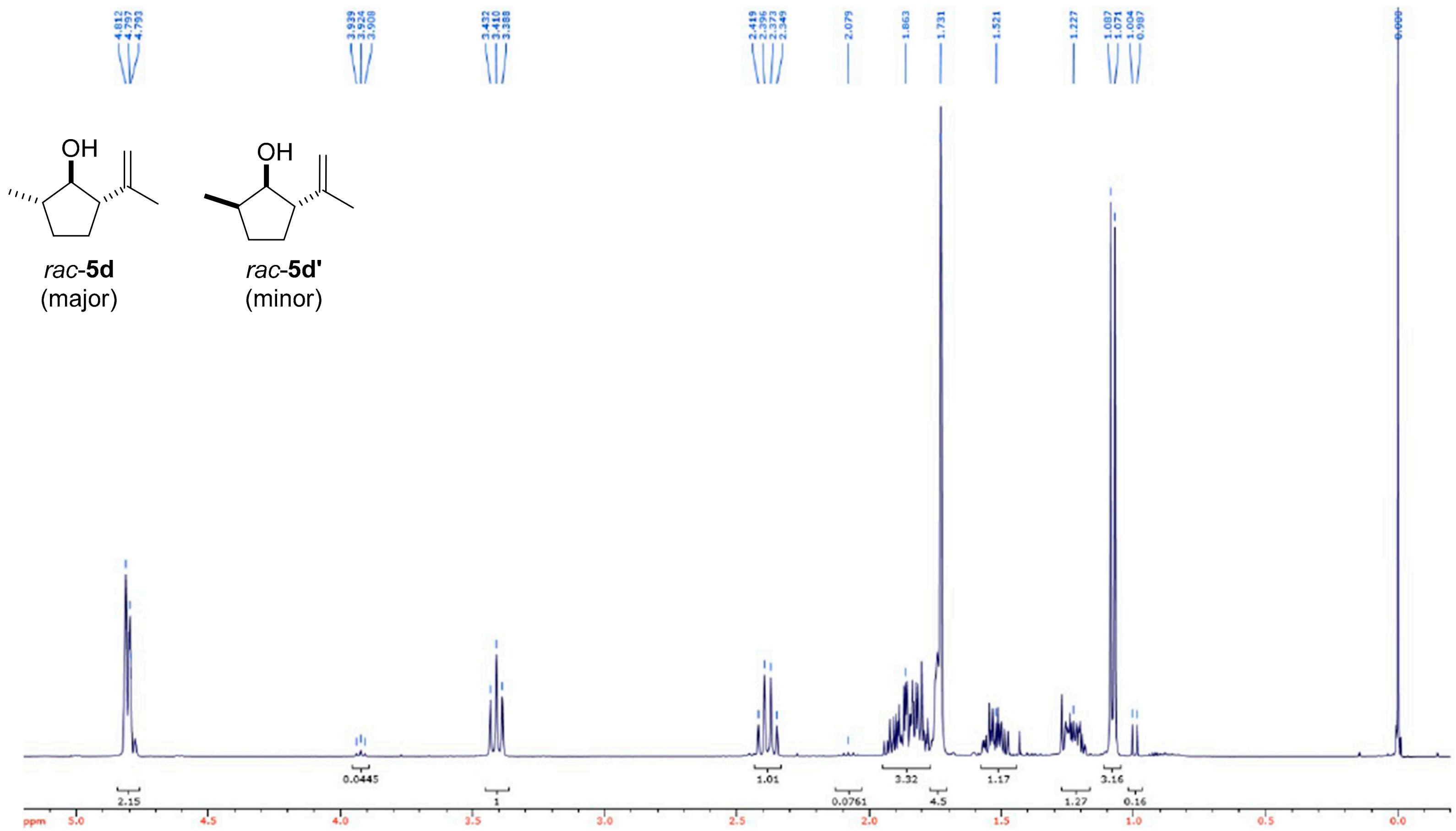

Figure S5. ^{1}H-NMR of **5d**/**d**′.

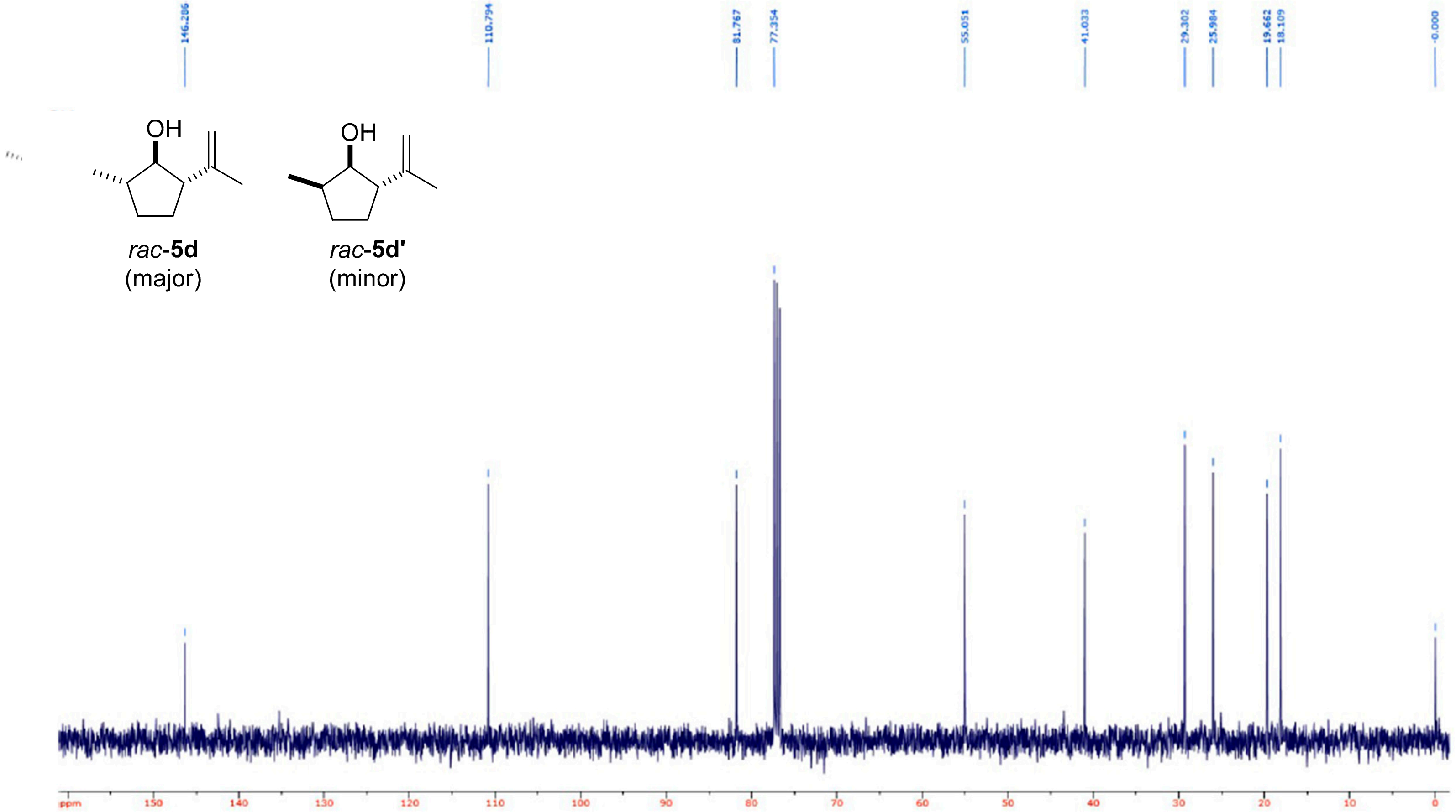

Figure S6. ^{13}C-NMR of **5d/d′**.
